# Fractal and Multifractal Analysis of PET-CT Images for Therapy Assessment of Metastatic Melanoma Patients under PD-1 Inhibitors: A Feasibility Study

**DOI:** 10.3390/cancers13205170

**Published:** 2021-10-15

**Authors:** Anastasia Kosmou, Christos Sachpekidis, Leyun Pan, George K. Matsopoulos, Jessica C. Hassel, Antonia Dimitrakopoulou-Strauss, Astero Provata

**Affiliations:** 1 Institute of Nanoscience and Nanotechnology, National Center for Scientific Research “Demokritos”, 15341 Athens, Greece; anast.kosmou@gmail.com; 2 School of Electrical and Computer Engineering, National Technical University of Athens, 15780 Athens, Greece; gmatso@esd.ece.ntua.gr; 3 Clinical Cooperation Unit Nuclear Medicine, German Cancer Research Center, 69120 Heidelberg, Germany; c.sachpekidis@dkfz-heidelberg.de (C.S.); l.pan@dkfz-heidelberg.de (L.P.); ads@ads-lgs.de (A.D.-S.); 4 Department of Dermatology and National Center for Tumor Disease, University Hospital Heidelberg, 69120 Heidelberg, Germany; Jessica.Hassel@med.uni-heidelberg.de

**Keywords:** metastatic melanoma, PET-CT imaging, nivolumab, pembrolizumab, ipilimumab, fractal dimensions, multifractal spectrum

## Abstract

**Simple Summary:**

The fractal dimension (FD) and the multifractal spectrum (MFS) are nonlinear quantitative measures which express the heterogeneity in the distribution of the tracer, F-18-fluorodeoxyglucose, (^18^F-FDG), in the body of patients suffering from metastatic melanoma. Given the well-documented, high accumulation of the tracer in tumor/metastatic sites, the measures expressing the tracer distribution also express the extent of metastases in the body. As such, FD and MFS can be employed to detect the presence of melanoma and to monitor the therapeutic outcome using the PET-CT follow-up digitized scans of the patients. In the present study, the FD and MFS measures of patients are evaluated before and during treatment with PD-1 inhibitors and are compared with the corresponding values of healthy controls. The MFS predictions agree with the PET Response Evaluation Criteria for Immunotherapy (PERCIMT) in 81% of the cases, while the FD agrees in 77% of all cases. Therefore, the quantitative MFS is proposed as an additional, alternative biomarker for monitoring the immunotherapy outcome in melanoma patients, after treatment with PD-1 inhibitors.

**Abstract:**

Longitudinal whole-body PET-CT scans with F-18-fluorodeoxyglucose (^18^F-FDG) in patients suffering from metastatic melanoma were analyzed and the tracer distribution in patients was compared with that of healthy controls. Nineteen patients with metastatic melanoma were scanned before, after two and after four cycles of treatment with PD-1 inhibitors (pembrolizumab, nivolumab) applied as monotherapy or as combination treatment with ipilimumab. For comparison eight healthy controls were analyzed. As quantitative measures for the comparison between controls and patients, the nonlinear fractal dimension (FD) and multifractal spectrum (MFS) were calculated from the digitized PET-CT scans. The FD and MFS measures, which capture the dispersion of the tracer in the body, decreased with disease progression, since the tracer particles tended to accumulate around metastatic sites in patients, while the measures increased when the patients’ clinical condition ameliorate. The MFS measure gave better predictions and were consistent with the PET Response Evaluation Criteria for Immunotherapy (PERCIMT) in 81% of the cases, while FD agreed in 77% of all cases. These results agree, qualitatively, with a previous study of our group when treatment with ipilimumab monotherapy was considered.

## 1. Introduction

Recent advances in immunotherapy have revolutionized cancer therapy and offer the possibility to activate the immune system against cancer cells. In particular, the use of immune checkpoint inhibitors (ICI) showed a dramatic improvement in the management of patients with metastatic melanoma leading in higher response rates and a prolongation of patient survival [1,2]. ICI treatment started with the use of the cytotoxic T-lymphocyte-associated protein 4 (CTLA-4) inhibitor ipilimumab, an antibody with response rates between 10% and 15% and long-term survival of approximately 20% [3,4]. In 2014, two programmed cell death protein (PD-1) inhibitors, pembrolizumab and nivolumab, were approved by the Food and Drug Administration and were then used as a monotherapy or in combination with ipilimumab. Larkin et al. reported on a median overall survival of more than 60.0 months for the combined use of nivolumab plus ipilimumab as compared with 36.9 months for nivolumab monotherapy and 19.9 months for ipilimumab monotherapy. Overall, survival at 5 years was 52% for the combination of nivolumab and ipilimumab, 44% for nivolumab and 26% for ipilimumab [5].

Despite these advances in melanoma treatment, a significant percentage of patients—approximately 40–60%—do not respond to ICI [2]. Furthermore, ICI treatment demonstrates different response patterns than conventional chemotherapy due to the fact that inflammation is generated through the T-cell activation rather than tumor lysis. In particular, ICI treatment can lead to atypical response patterns, like pseudoprogression (initial increase in tumor burden followed by a decrease), hyperprogression (rapid disease progression with very poor survival), dissociated response (regression of some lesions and appearance of new lesions) and late response after therapy discontinuation. All these challenges underline the need for dedicated biomarkers that can reliably and early serve ICI treatment monitoring on a personalized basis. Imaging, in particular with hybrid techniques, like PET-CT plays an important role for immunotherapy monitoring [6].

For the assessment of the therapeutic outcome of ICI therapy ^18^F-FDG PET-CT can be used with good results [7,8,9,10,11,12,13]. For that purpose, a baseline PET-CT study as well as follow-up studies during treatment need to be obtained. The traditional evaluation of the PET images is based on standard Volume of Interest (VOI) based techniques and on the calculation of standardized uptake values (SUV) in the tumors and reference areas [14]. New approaches include not only linear measures of tracer uptake, like SUV calculations, but also nonlinear ones, like fractal dimensions-FD, correlation dimensions -CD, and multifractal spectrum—MFS or combinations of them [15,16,17].

In a previous analysis of our group, we studied a cohort of 31 patients suffering from metastatic melanoma under treatment with ipilimumab monotherapy [17]. The main outcome of that study was that the fractal dimension and the multifractal moments decrease as the metastases progress in the body of the patient in terms of nonresponse. The explanation of this effect is the following: In absence of metastases the tracer particles spread homogeneously in the body and thus the tracer covers uniformly the 3D space of the body (with few exceptions related to regular uptake from organs). When tumorous lesions are present, the tracer concentrates in a subspace of the 3D space, and the fractal and multifractal measures capture exactly the dimensionality of the subspace where the tracer is dispersed. Because the cancerous cells, as well as the tracer particles, travel through the body via the circulatory or the lymphatic system, it is expected that the fractal dimension **Df** of the metastatic melanoma, in its most extensive form, could reach values close to **Df** = 2.7, which are the fractal dimensions of the circulatory system as already reported in 1977 by Mandelbrot [15] and others [18,19,20,21,22].

In the present study the above ideas are further evaluated using a new cohort of patients under ICI treatment with different monoclonal antibodies, namely the PD-1 inhibitors nivolumab and pembrolizumab as well as the combination of nivolumab and ipilimumab. We use the FD and MFS nonlinear measures to record the extent of metastatic activity and evolution in melanoma patients during the stages of treatment with the different monoclonal antibodies. The current results agree qualitatively and qualitatively with the previous study where only ipilimumab monotherapy was employed. Namely, an increase of the FD and MFS measures indicates clinical amelioration of the patient while a decrease of the measures indicates clinical deterioration. The FD and MFS measures can then be used as additional biomarkers for monitoring the evolution of metastatic melanoma, independently of the medical treatment with the different monoclonal antibodies. Because of their high degree of correct evaluation of the melanoma evolution, it is proposed that the nonlinear measures can be used, on the one hand by the clinicians for taking into account in their assessments and, on the other hand, they can be added in the list of implemented biomarkers to increase of prediction ability of artificial intelligence (AI) medical evaluation algorithms.

The presentation of the work is organized as follows. In Section 2.1, the PET-CT data of all patients are presented, before and during treatment, together with their outcome according to the PET Response Evaluation Criteria for Immunotherapy (PERCIMT) [8]. In the same section, the PET-CT data of the healthy controls are presented for comparative purposes. In Section 2.2, the definition of the FD and MFS measures as applied to the digitized PET-CT images are presented. Specific details on the FD and MFS measures and their calculations are provided in Appendix A, while particular examples are discussed in Appendix B and Appendix C. In Section 3, the results are presented. In particular, the FD and MFS measures of all controls and all patients at the different follow-up scans are reported. Moreover, the FD and MFS predictions are evaluated in comparison with both the patient outcome according to PERCIMT and the respective results derived from the controls. In Section 4, the impact of immune-related adverse events (irAEs) in PET-CT is discussed. Particular attention was paid to ICI-related colitis, since it may often cause diffuse ^18^F-FDG accumulation in the colon leading to false positive results in FD and MFS measures [17]. In the Discussion section, a previous study of metastatic melanoma and other related works are compared with the present approach and open problem are presented. Finally, in the Conclusions, the main results of this study are recapitulated.

## 2. Materials and Methods

In the next Section 2.1, the PET-CT scans of patients and controls are presented, while in Section 2.2, the clinical evaluation of the patients is assessed in baseline and two follow-up PET-CT examinations. PET signs suggestive of irAEs as well as causes of non-specific tracer uptake are also discussed. In Section 2.3, the nonlinear FD and MFS measures are briefly presented and their implementation regarding the quantitative description of the extent of metastases is described.

### 2.1. The PET-CT Data

Nineteen patients, indexed as P1, P2, P19, suffering from metastatic melanoma participated in this study. The age and sex of each patient are listed in Table 1, column 2. Each patient was scanned before treatment (baseline study), after two cycles of treatment with anti PD-1 antibodies (interim study) and once after four cycles of treatment (final study), see Table 1, column 3. Pembrolizumab was administered intravenously at a dose of 2 mg/kg every 3 weeks, and nivolumab was administered intravenously at a dose of 3 mg/kg every 2 weeks. The combination ICI therapy was administered as an induction of 4 cycles of nivolumab (1 mg/kg) and ipilimumab (3 mg/kg) every 3 weeks, followed by single agent nivolumab administration (3 mg/kg) every 2 weeks. The included patients had not received chemotherapy for at least 1 month prior to the initial PET-CT studies. None of the patients had a history of diabetes. Patients gave written informed consent to participate in the study and to have their medical records released. The study was approved by the Ethical Committee of the University of Heidelberg and the Federal Agency for Radiation Protection (Bundesamt für Strahlenschutz). Part of these patients have been published elsewhere under different aspects [9,10].

Patients underwent a whole-body PET-CT after intravenous administration of maximum 250 MBq ^18^F-FDG 60 min post-injection (p.i.). Imaging was performed from the head to the feet with an image duration of 2 min per bed position. A dedicated PET-CT system (Biograph mCT, S128, Siemens Co., Erlangen, Germany) with an axial field of view of 21.6 cm with TruePoint and TrueV, operated in a three-dimensional mode was used. A low-dose attenuation CT (120 kV, 30 mA) was used for attenuation correction of the PET data and for image fusion. All PET images were attenuation-corrected and an image matrix of (400 × 400) pixels was used for iterative image reconstruction. Iterative image reconstruction was based on the ordered subset expectation maximization (OSEM) algorithm with two iterations and 21 subsets as well as time of flight (TOF).

The patient outcome according to PERCIMT at the follow-up examinations is reported in the last column of Table 1. In particular, patient responses to ICI were classified as following: CMR: Complete Metabolic Response; PMR: Partial Metabolic Response; SMD: Stable Metabolic Disease; PMD: Progressive Metabolic Disease. The treatment is indicated for each patient in column 4 of the same Table. PET signs suggestive of irAEs as well as causes of non-specific tracer uptake are reported in column 5. Particular attention will be devoted in Section 4 in the case of colitis since it leads to false positive results in blind calculation of the fractal and multifractal measures, as will be explained in Section 4.

For comparative reasons the PET-CT scans of 8 healthy controls are also presented at the end of the same table. The controls are indexed H1, H2, H8. Only a single PET-CT study of the controls is available.

### 2.2. Clinical Evaluation and Radiological irAEs

The PET-CT evaluation of the patients was based on the visual and quantitative evaluation of the images by two experienced nuclear medicine physicians (CS, ADS). The clinical outcome data regarding response to therapy were based on the PERCIMT criteria [8].

On the 5th column of Table 1, PET signs of irAEs as well as causes of non-specific tracer accumulation are presented, separately, for each patient. With regard to irAEs, these included radiologic signs of thyroiditis, colitis, bone marrow activation, arthritis, duodenitis, to list the most frequent ones. Among them, colitis is known to heavily affect the diagnostic ability of the FD and MFS measures. In the presence of radiologic colitis, the tracer demonstrates a diffuse accumulation in the colon, comparable to the accumulation in the metastases. For this reason, in the 2nd part of the study, Section 4, the cases of colitis are excluded from the statistics, since they always lead to false positive results due to immune related adverse effects.

High tracer uptake, irrespective to the presence of tumorous lesions, is also recorded in the brain. To avoid this effect the brain regions are excluded from the calculations in all subjects and studies and only the areas from nose to toes are considered.

### 2.3. The Fractal Dimension and the Multifractal Spectrum

The PET-CT images of the patients demonstrate the dispersion of the tracer ^18^F-FDG in the body and are valuable for tumor detection and staging since ^18^F-FDG has the tendency to concentrate in tumor lesions. In clinical practice, ^18^F-FDG images are evaluated visually and quantitatively using mostly the calculation of SUVs. The FD and MFS give additional quantitative measures of the tracer extension in the body, which also mirrors the lesion extension. Based on this alternative approach, one may evaluate the clinical deterioration or amelioration of the patient’s response to treatment, depending on the change of the FD and MFS measures of the patient.

To calculate the FD and the MFS we first digitize the PET-CT images extracting a 3D matrix which accounts for the local concentration of the tracer in the body. To this end, the Insight Segmentation and Registration Toolkit (ΙΤΚ) was used to transform the DICOM images extracted from the PET-CT scans into a 3D tracer concentration array C. The C-array comprises of 400 × 400 pixels for each planar scan and between 370–430 (without the brain) scans along the axial direction, depending on the patient. Approximately, each C-matrix comprises of 400 × 400 × 400 voxels. The voxel size then is 2 mm × 2 mm × 4 mm and this is the minimum elemental size used in the fractal and multifractal analyses. Besides the digitization with the ITK toolkit, we also used the package ImageJ to reduce the DICOM images to the ASCII form needed for the C-arrays (results not shown).

The ^18^F-FDG concentration ***C*(*i,j,k*)** in box at position **(*i,j,k*)** takes values **0** **≤** ***C*(*i,j,k*)** **≤** **255.** In some cases, a threshold **w** is set on the concentration, which accounts for meaningful recording of ^18^F-FDG above the threshold and negligible concentration below it. In the present study, the threshold **w = 1** was used, in order to take into account even the smallest ^18^F-FDG concentrations which could account for very small metastatic lesions. This threshold gave the best results and was the most consistent with the medical records.

Based on the ***C*(*i,j,k*)** matrix which is directly extracted from the PET-CT data of the patients and controls, we apply the box-counting method to calculate the FD and MFS measures. The box counting technique consists in segmenting the 3D space of the body in boxes of different sizes **s**, and to calculate the number of boxes which contain ^18^F-FDG, as a function of **s**. A power law fitting to this data allows to directly extract the fractal dimension FD, as the exponent **Df** of the fit. The value of **Df** accounts for the fraction of the body which is covered by the ^18^F-FDG. For healthy controls **Df** takes high values <3. When **Df** increases between treatments then an improvement of the patient’s condition takes place (e.g., number and/or extension of metastatic sites decrease), whereas if **Df** decreases then the patient’s condition deteriorates (increased metastatic activity). The precise definition and calculation of the FD measure and details on the computation of **Df** are provided in Appendix A.

**Figure 1:**
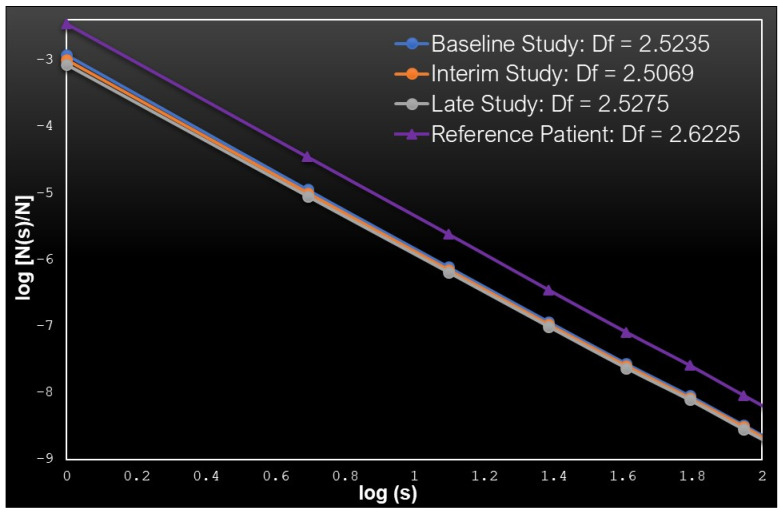
The normalized number of boxes **N(s)/N** which contain the tracer as a function of the box size **s** in a double logarithmic scale. The blue line corresponds to the baseline study of patient P9, the orange line to the interim study and the gray line to the final study of the same patient. For comparison the purple line representing healthy control H3 is also depicted.

Typical examples of FD calculations are shown in Figure 1. In this figure **N(s)** accounts for the number of 3D boxes of size **s** which contain the tracer, while **N** is the total number of boxes used in the calculations.

Figure 1 depicts the normalized number of boxes **N(s)/N** as a function of the box size **s**, in a double logarithmic scale. The results concern Patient P9 at the three studies. The reference control H3 is also added, for comparison. The **Df** values of patient and control are reported on the figure. Note that the **Df** of the control is higher than the **Df** of the patient. This is consistent with the fact that the tracer splits more homogeneously in the 3D space of the control’s body, since there are no lesions which cause local concentrations of the tracer. Only specific organs are accumulating some amount of the tracer and that is why the calculated **Df** drops below 3 (takes value **Df** = 2.6225) even for the reference subjects. We note that the **Df** value for the patient, whose state is characterized as stable SMD, is consistently lower than the reference in all studies, and stays close to **Df** = 2.5. This means that the tracer is attracted and concentrates in the lesions, which cover a subspace lower than fractal dimensions in the case of the healthy control. Similar typical images for the cases of PMR and PMD are shown in Appendix B.

To present quantitative results on the clinical amelioration or deterioration of the patients’ conditions after treatment, we compare the FD values at the interim **(j = 2)** and final **(j = 3)** stages with the baseline stage **(j = 1)**. Namely, if the fractal dimensions of the interim study **Df**(**j = 2)** or late study **Df**(**j = 3)** after ICI treatments are greater than the baseline study **Df(j = 1)**, the patient’s improvement is recorded. In the opposite case, **Df(j = 1)** is greater than **Df(j = 2)** or **Df(j = 3)**, patient’s deterioration is recorded. Finally, if **|Df(j = 2) − Df(j = 1)|** **< 0.02 or |Df(j = 3) − Df(j = 1)| < 0.02,** patient’s condition can be considered as stable between stages. The FD results on the patients and controls are presented in Section 3 and Table 2.

Following the calculation of the FD measure, for the MFS measure the box-counting technique is also employed. After the segmentation of the body in boxes of size **s**, the moments ***Dq*** of integer order **q,** of the tracer distribution are calculated (**q** can take positive and negative values). The different **q**-moments provide details of the tracer dispersion in the body. Namely, the negative **q**-values represent the extent of small tracer concentrations, while the positive **q**-values correspond to high tracer concentrations. The precise definition of the moments and their calculation is provided in Appendix A.

**Figure 2:**
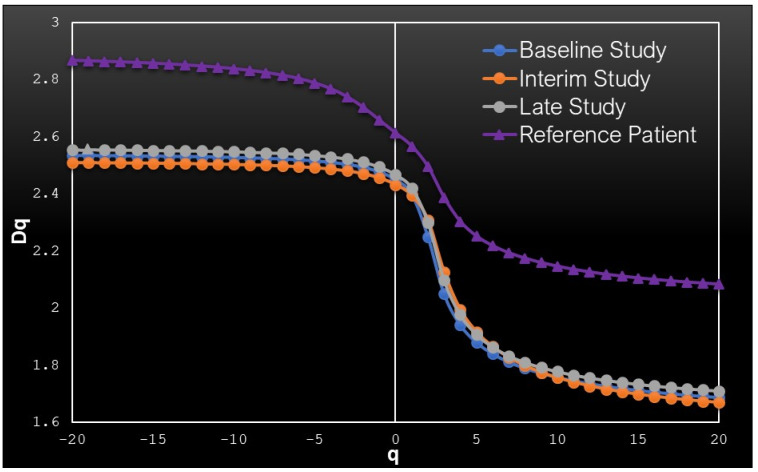
Typical MFS spectrum of patient with SMD. The blue line corresponds to the baseline study of patient P12, the orange line corresponds to the interim study and the gray line to the final study of the same patient. For comparison, the purple line representing healthy control H5 is also depicted.

A typical MFS spectrum is provided in Figure 2, for **−20 ≤ q ≤ +20**. Here, the MFS spectrum of the reference control lies above those of the patient, similarly to FD behavior. The spectra of the patient are placed on top of one another, indicating a stable disease, without major influence by the treatment for the case of patient P12. Other typical MFS spectra of patients with PMR and PMD are presented in Appendix C.

It is worth noting here that the negative q-orders are enhanced by the very small (infinitesimal) local concentrations, which are accentuated when exponentiated on a negative power (see Appendix A). Therefore, negative **q-**exponents are important for detecting small tracer concentrations, which usually indicate the presence of small-size metastases, possibly non-visible by eye. On the other hand, large tracer concentrations are accentuated by positive **q-**orders, indicating either concentrations around tumors of large sizes, or concentrations around organs which accumulate ^18^F-FDG.

Regarding the presence of ^18^F-FDG in specific organs, the tracer also accumulates in certain healthy tissues, which consume glucose, like the brain or the liver, and this should be considered in the evaluation. Note that the brain is already excluded, as discussed at the end of Section 2.2. Moreover, ^18^F-FDG is normally excreted by the urinary tract, leading to depiction of the kidneys, ureters and the urinary bladder. That is why it is useful to study the ^18^F-FDG dispersion comparatively, between patients and controls, since they all involve consumption of ^18^F-FDG in the healthy organs, but in additions the patients present further concentration in the metastatic lesions.

While it is instructive to show the difference between healthy controls and patients at the different q-moments, as in Figure 2, it is also useful to define a unique, cumulative multifractal measure of clinical improvement/deterioration based on all moments. This cumulative measure **Δ*D*(*j*)** takes into account the difference of the moments ***Dq*** of the patient at stage **j**, from the baseline **j = 1,** $\left[D_{q}^{j}-D_{q}^{1}\right]$. These differences are averaged over many values of q, and the measure **Δ*D*(*j*)** is normalized. In the present study we average over 21 moments, **−10** **≤** **q** **≤** **10,** and we normalize accordingly as: $\Delta D\left(j\right)=\overset{10}{\underset{q=-10}{\sum }}\left[D_{q}^{j}-D_{q}^{1}\right]/21$**.** Further details on this cumulative measure are also provided in the Appendix A.

## 3. Results

In this section, first the calculations of the FD are presented in subSection 3.1, followed by the calculations of the MFS in subSection 3.2. The results are briefly discussed at the end of each subsection, while a more extensive discussion follows in Section 5.

### 3.1. Fractal Dimensions

The results on the FD for patients and controls are recapitulated in Table 2. In the 1st column the order number of the patients is shown in order to match with the data on Table 1. On the second column the study stage is indicated for each patient. On column 3, the FD of each patient calculated from the PET-CT data with **w = 1**, is recorded. The PET-irAEs and causes of non-specific tracer uptake, which may account for false positive/negative results, are provided in column 4, while the clinical evaluation of each patient follow-up PET-CT scans as a response to ICI is reported in column 5. The matching between clinical evaluation and FD results is indicated in the last column (YES = matching, NO = not matching).

For the controls, which are listed at the end of Table 2, only the fractal dimensions at the baseline (unique) study are recorded, together with their side effects.

From the above results, the FD method matches the clinical results in 71.05% of the cases if the side-effects are not taken into account. Among the side-effects, the one which predominantly affects the tracer dispersion is colitis (see Table 1). If the cases where colitis is present are excluded, then the matching increases to 77.42%.

Apart from the fractal dimensions based on the box-counting method, the correlation dimensions were also estimated, and the results (not shown) were in agreement with the FD conclusions in Table 2.

### 3.2. Multifractal Spectrum

Similar to the results on the FD presented in Section 3.1, here, the results on the MFS are recapitulated in Table 3. On the 3rd column of this table, the average multifractal index, **<MFS>_j,_** and the cumulative multifractal measure **ΔD(j)** are recorded of each patient and stage. For the calculations of these quantities see Section 2.3 and Appendix A. The PET-irAEs and causes of non-specific tracer uptake, as well as the patient’s clinical outcome are provided in columns 4 and 5, respectively. The matching between clinical evaluation and MFS results is indicated in the last column. For the controls listed at the end of Table 3, only the **<MFS>_1_** is calculated.

From the calculations of the MFS, the results match the clinical evaluation in 76.32% of the cases if the side-effects are not taken into account. If the cases of colitis (see Table 1) are excluded, then the matching increases to 80.65%. In both cases, with or without excluding colitis, the MFS confirms better the clinical evaluation than the FD measure.

## 4. Side-Effects and the Case of Colitis

**Figure 3:**
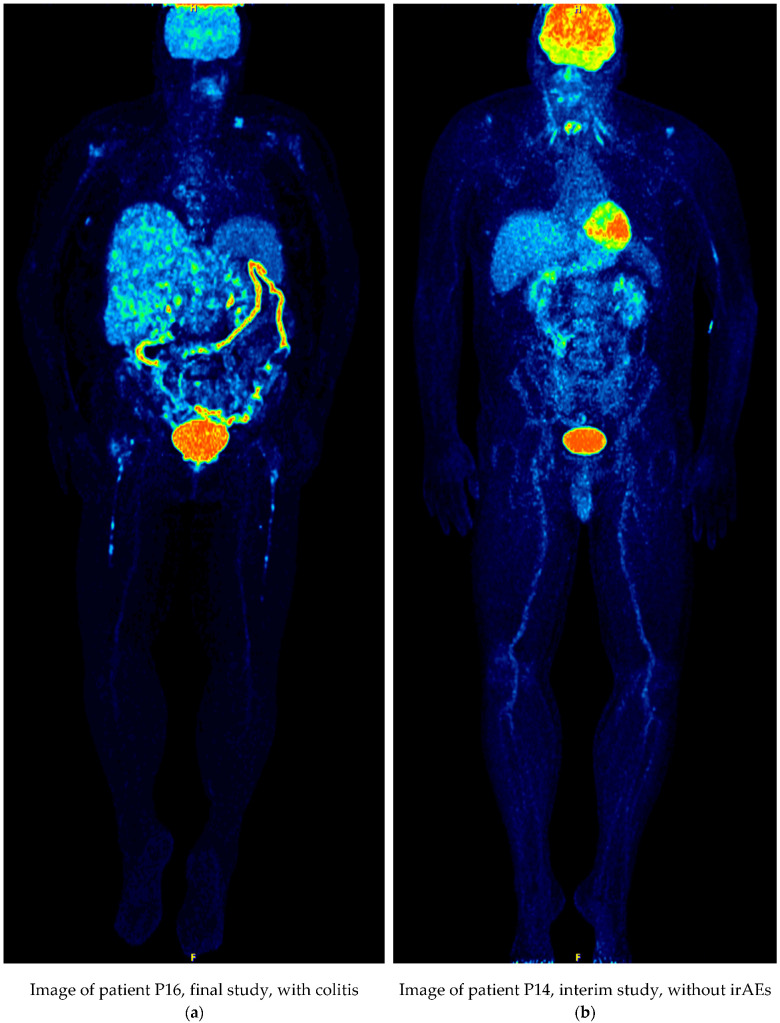
MIP PET-CT images for patients with metastatic melanoma: (**a**) Patient P16 at final stage with signs of colitis; (**b**) Patient P14 at interim stage without irAEs effects.

As discussed in the previous section, the presence of colitis perturbs the outcome of the FD and MFS measures because the tracer concentrates in the colon in addition to the accumulations on the metastases. In Figure 3a the PET-CT image of a metastatic melanoma patient with abnormal tracer uptake in the colon (P16) is depicted. For comparison, in Figure 3b the case of a patient (P14) with metastatic melanoma without radiological signs of side-effects as a response to the treatment is shown.

Visual inspection of Figure 3a, (Patient P16) reveals increased uptake of the tracer in the colon area to a large extent, as compared to the Patient P14, in Figure 3b, where the ^18^F-FDG concentrates only in organs and metastatic lesions. When the FD and MFS measures are blindly applied, the uptake colon areas in Patient P16 are mistakenly considered as metastatic tissue, giving rise to false positive results. In this patient in the interim and final studies, the tracer spreads thoroughly in the body because of the colitis and as a result the FD and MFS measures increase, falsely indicating that the patients’ condition clinically ameliorates.

This is the reason why the results in Section 3 considerably improved when the cases of patients/stages with colitis were excluded from the statistics. As seen from Table 1 and Table 2, colitis was a side effect in 8/66 studies in all patients and controls.

Other side-effects, such as thyroiditis, arthritis, muscular uptake and others (see Table 1), do not substantially contribute to the dispersion of the tracer and, to a good approximation, can be ignored.

## 5. Discussion

In a previous study of our group [17], a different cohort of patients suffering from metastatic melanoma and controls were investigated. The cohort consisted of 2 healthy controls and 31 patients. The patients received four cycles of treatment with the monoclonal antibody ipilimumab as monotherapy and they were scanned at baseline, after two and after four treatment cycles. A similar fractal and multifractal analysis of the patients produced results comparable to the ones presented here. Namely, in 83% of the cases the nonlinear analysis results matched with the clinical outcome. Note that in [17] a different notation related to the outcome of the PET-CT visual interpretation was used: PR for partial remission, SD for stable disease, PD for progressive disease and MR for mixed response.

The outcome of the present study together with the previous results by Breki et al. [17], indicate that the nonlinear measures can be useful as biomarkers for metastatic cancers because they accentuate even the smallest local uptakes of the tracer (^18^F-FDG) on metastases of tiny sizes, not visible with the naked eye.

Further improvements of this method include:

1. The computational exemption of organs, which accumulate or excrete the tracer. This is a major weakness of the present methods, and if solved, we could expect matching evaluations in over 95% of the cases. Possible methods from the domain of artificial intelligence may soon offer a solution to this problem.
2. The development of novel, more specific radiotracers which only accumulate to a markedly higher extent in the tumorous lesions rather than in the physiological tissues. Similarly to the previous case, we expect an important improvement of the matching between clinical evaluation and fractal/multifractal analysis if the non-pathological accumulation of the tracer can be excluded in one or the other way.
3. The use of PET-CT images of higher resolution. Such images could offer the possibility to extract more accurate FD and MFS measures. These measures in combination with conventional measures such as SUV, metabolic tumor volume (MTV), total lesion glycolysis (TLG) as well as the use of artificial intelligence (AI) algorithms for image segmentation may help to achieve a more precise evaluation of immunotherapy treatment response in the future.

## 6. Conclusions

The fractal dimensions and the multifractal spectrum of the ^18^F-FDG tracer dispersion were studied in patients with metastatic melanoma before and after treatment with PD-1 inhibitors as monotherapy or in combination with the CTLA-4 inhibitor ipilimumab. The tracer distribution was extracted from the PET-CT scans of the patients after two and four cycles of treatment with nivolumab or pembrolizumab monotherapy or a combination of nivolumab and ipilimumab. It was shown that, if the fractal dimension and the average multifractal index are employed as biomarkers to assess the patient condition and the evolution of the disease, the matching with the clinical outcome is 77% when the FD measure is used and increases to 81% when the MFS measure is considered. The diagnostic ability of these nonlinear measures can further increase if the physiological uptake of ^18^F-FDG by the organs as well as unspecific ^18^F-FDG uptake due to immune related effects, like colitis, are excluded.

The present results agree with previous studies in patients treated solely with ipilimumab [17]. Namely, the fractal dimensions and the average multifractal index decrease as the disease progresses and the number of metastases increases. Based on these findings the FD and MFS measures can be used as promising computational biomarkers for diagnosis of metastatic cancers and for monitoring the therapeutic result of ICI treatment. These preliminary results should be evaluated in larger patient cohorts prospectively.

## Figures and Tables

**Table 1:** Presentation of patient and control data and clinical evaluation.

Patients	Age/Sex	Study	Medication	PET-irAEs and Causes of Non-Specific Tracer Uptake	PET-CT Evaluation (PERCIMT)
P1	56/F	Baseline	Ipilimumab/Nivolumab		
		Interim		thyroiditis	PMD
		Final		colitis	PMD
P2	48/F	Baseline	Ipilimumab/Nivolumab		
		Interim		radiopharmaceutical uptake in the injection site	SMD
		Final		radiopharmaceutical uptake in the injection site	CMR
P3	60/F	Baseline	Ipilimumab/Nivolumab		
		Interim		radiopharmaceutical uptake in the injection site	PMR
		Final		thyroiditis, colitis, bone marrow activation	PMR
P4	52/M	Baseline	Pembrolizumab	muscle uptake	
		Interim		arthritis, muscle uptake	SMD
		Final		arthritis	SMD
P5	46/F	Baseline	Ipilimumab/Nivolumab	radiopharmaceutical uptake in the injection site	
		Interim		duodenitis, muscle uptake	SMD
		Final		duodenitis, colitis	PMD
P6	68/M	Baseline	Pembrolizumab		
		Interim			SMD
		Final			PMR
P7	44/F	Baseline	Ipilimumab/Nivolumab		
		Interim		colitis, bone marrow activation	PMR
		Final		bone marrow activation	PMR
P8	50/F	Baseline	Ipilimumab/Nivolumab	laryngeal uptake	
		Interim		bone marrow activation	SMD
		Final		arthritis, bone marrow activation, radiopharmaceutical uptake in the injection site, muscle uptake	CMR
P9	55/F	Baseline	Nivolumab	bone marrow activation	
		Interim		bone marrow activation	SMD
		Final		bone marrow activation	SMD
P10	54/M	Baseline	Ipilimumab/Nivolumab		
		Interim			PMR
		Final			PMR
P11	20/F	Baseline	Ipilimumab/Nivolumab	bone marrow activation	
		Interim			PMD
		Final			PMD
P12	84/F	Baseline	Pembrolizumab		
		Interim			SMD
		Final		muscle uptake	SMD
P13	53/F	Baseline	Nivolumab	bone marrow activation	
		Interim			SMD
		Final			SMD
P14	52/M	Baseline	Pembrolizumab	laryngeal uptake	
		Interim		laryngeal uptake	PMR
		Final		laryngeal uptake, radiopharmaceutical uptake in the injection site	PMR
P15	52/M	Baseline	Pembrolizumab		
		Interim			SMD
		Final		signs of colitis in descending colon	SMD
P16	71/F	Baseline	Ipilimumab/Nivolumab	colon uptake	
		Interim		colitis, sarcoid-like mediastinal lymphadenopathy	PMD
		Final		colitis	PMD
P17	34/F	Baseline	Ipilimumab/Nivolumab		
		Interim		bone marrow activation, colitis, muscle uptake	CMR
		Final		brown fat activation	CMR
P18	78/M	Baseline	Pembrolizumab	radiopharmaceutical uptake in the injection site	
		Interim		arthritis	PMD
		Final			PMD
P19	59/M	Baseline	Ipilimumab/Nivolumab		
		Interim		sarcoid-like mediastinal lymphadenopathy, muscle uptake	PMR
		Final		sarcoid-like mediastinal lymphadenopathy	PMR
HEALTHY CONTROLS					
H1	49/M		-	muscle uptake	
H2	63/M		-	radiopharmaceutical uptake in the injection site	
H3	39/M		-	-	
H4	61/M		-	-	
H5	52/M		-	-	
H6	63/M		-	muscle uptake	
H7	69/M		-	-	
H8	60/M		-	-	

**Table 2:** Presentation of patient and control Fractal Dimensions (FD).

Patients	Study	FD	Side Effects	Clinical Outcome	Matching
P1	Baseline	2.546			
	Interim	2.459	thyroiditis	PMD	YES
	Final	2.558	colitis	PMD	NO
P2	Baseline	2.523			
	Interim	2.515	radiopharmaceutical uptake in the injection site	SMD	YES
	Final	2.476	radiopharmaceutical uptake in the injection site	CMR	NO
P3	Baseline	2.537			
	Interim	2.557	radiopharmaceutical uptake in the injection site	PMR	YES
	Final	2.527	thyroiditis, colitis, bone marrow activation	PMR	NO
P4	Baseline	2.566	muscle uptake		
	Interim	2.543	arthritis, muscle uptake	SMD	YES
	Final	2.543	arthritis	SMD	YES
P5	Baseline	2.487	radiopharmaceutical uptake in the injection site		
	Interim	2.497	duodenitis, muscle uptake	SMD	YES
	Final	2.448	duodenitis, colitis	PMD	YES
P6	Baseline	2.519			
	Interim	2.530		SMD	YES
	Final	2.503		PMR	NO
P7	Baseline	2.542			
	Interim	2.544	colitis, bone marrow activation	PMR	YES
	Final	2.562	bone marrow activation	PMR	YES
P8	Baseline	2.544	laryngeal uptake		
	Interim	2.399	bone marrow activation	SMD	NO
	Final	2.476	arthritis, bone marrow activation, radiopharmaceutical uptake in the injection site, muscle uptake	CMR	NO
P9	Baseline	2.524	bone marrow activation		
	Interim	2.507	bone marrow activation	SMD	YES
	Final	2.528	bone marrow activation	SMD	YES
P10	Baseline	2.534			
	Interim	2.562		PMR	YES
	Final	2.574		PMR	YES
P11	Baseline	2.499	bone marrow activation		
	Interim	2.398		PMD	YES
	Final	2.474		PMD	YES
P12	Baseline	2.513			
	Interim	2.480		SMD	NO
	Final	2.537	muscle uptake	SMD	YES
P13	Baseline	2.603	bone marrow activation		
	Interim	2.596		SMD	YES
	Final	2.590		SMD	YES
P14	Baseline	2.554	laryngeal uptake		
	Interim	2.518	laryngeal uptake	PMR	NO
	Final	2.557	laryngeal uptake, radiopharmaceutical uptake in the injection site	PMR	YES
P15	Baseline	2.556			
	Interim	2.604		SMD	NO
	Final	2.567	signs of colitis in descending colon	SMD	YES
P16	Baseline	2.398	colon uptake		
	Interim	2.511	colitis, sarcoid-like mediastinal lymphadenopathy	PMD	NO
	Final	2.574	colitis	PMD	NO
P17	Baseline	2.473			
	Interim	2.518	bone marrow activation, colitis, muscle uptake	CMR	YES
	Final	2.515	brown fat activation	CMR	YES
P18	Baseline	2.541	radiopharmaceutical uptake in the injection site		
	Interim	2.523	arthritis hip	PMD	YES
	Final	2.539		PMD	YES
P19	Baseline	2.549			
	Interim	2.572	sarcoid-like mediastinal lymphadenopathy, muscle uptake	PMR	YES
	Final	2.580	sarcoid-like mediastinal lymphadenopathy	PMR	YES
HEALTHY CONTROLS					
H1		2.544	muscle uptake		
H2		2.614	radiopharmaceutical uptake in the injection site		
H3		2.623	-		
H4		2.496	-		
H5		2.646	-		
H6		2.518	muscle uptake		
H7		2.581	-		
H8		2.589	-		

**Table 3:** Presentation of MFS and cumulative measure **ΔD(j)** results, for patients and healthy controls.

Patients	Study	<MFS>/ΔD(j)	Side Effects	Clinical Outcome	Matching
P1	Baseline	2.389			
	Interim	2.178/−0.211	thyroiditis	PMD	YES
	Final	2.460/0.071	colitis	PMD	NO
P2	Baseline	2.314			
	Interim	2.281/−0.033	radiopharmaceutical uptake in the injection site	SMD	YES
	Final	2.523/0.210	radiopharmaceutical uptake in the injection site	CMR	YES
P3	Baseline	2.341			
	Interim	2.358/0.017	radiopharmaceutical uptake in the injection site	PMR	YES
	Final	2.295/−0.046	thyroiditis, colitis, bone marrow activation	PMR	NO
P4	Baseline	2.472	muscle uptake		
	Interim	2.463/−0.009	arthritis, muscle uptake	SMD	YES
	Final	2.428/−0.044	arthritis	SMD	YES
P5	Baseline	2.248	radiopharmaceutical uptake in the injection site		
	Interim	2.485/0.237	duodenitis, muscle uptake	SMD	NO
	Final	2.202/−0.047	duodenitis, colitis	PMD	YES
P6	Baseline	2.450			
	Interim	2.399/−0.051		SMD	YES
	Final	2.252/−0.198		PMR	NO
P7	Baseline	2.309			
	Interim	2.460/0.151	colitis, bone marrow activation	PMR	YES
	Final	2.484/0.176	bone marrow activation	PMR	YES
P8	Baseline	2.306	laryngeal uptake		
	Interim	2.154/−0.152	bone marrow activation	SMD	NO
	Final	2.242/−0.064	arthritis, bone marrow activation, radiopharmaceutical uptake in the injection site, muscle uptake	CMR	NO
P9	Baseline	2.425	bone marrow activation		
	Interim	2.353/−0.072	bone marrow activation	SMD	YES
	Final	2.305/−0.120	bone marrow activation	SMD	YES
P10	Baseline	2.273			
	Interim	2.470/0.1907		PMR	YES
	Final	2.384/0.111		PMR	YES
P11	Baseline	2.259	bone marrow activation		
	Interim	2.145/−0.115		PMD	YES
	Final	2.237/−0.023		PMD	YES
P12	Baseline	2.242			
	Interim	2.243/0.001		SMD	YES
	Final	2.266/0.024	muscle uptake	SMD	YES
P13	Baseline	2.394	bone marrow activation		
	Interim	2.466/0.072		SMD	YES
	Final	2.461/0.067		SMD	YES
P14	Baseline	2.302	laryngeal uptake		
	Interim	2.511/0.209	laryngeal uptake	PMR	YES
	Final	2.310/0.008	laryngeal uptake, radiopharmaceutical uptake in the injection site	PMR	YES
P15	Baseline	2.233			
	Interim	2.376/0.143		SMD	NO
	Final	2.253/0.020	signs of colitis in descending colon	SMD	YES
P16	Baseline	2.155	colon uptake		
	Interim	2.261/0.106	colitis, sarcoid-like mediastinal lymphadenopathy	PMD	NO
	Final	2.378/0.223	colitis	PMD	NO
P17	Baseline	2.192			
	Interim	2.496/0.303	bone marrow activation, colitis, muscle uptake	CMR	YES
	Final	2.390/0.198	brown fat activation	CMR	YES
P18	Baseline	2.346	radiopharmaceutical uptake in the injection site		
	Interim	2.269/−0.077	arthritis hip	PMD	YES
	Final	2.413/0.066		PMD	NO
P19	Baseline	2.266			
	Interim	2.487/0.221	sarcoid-like mediastinal lymphadenopathy, muscle uptake	PMR	YES
	Final	2.346/0.080	sarcoid-like mediastinal lymphadenopathy	PMR	YES
HEALTHY CONTROLS					
H1		2.560	muscle uptake		
H2		2.365	radiopharmaceutical uptake in the injection site		
H3		2.442	-		
H4		2.322	-		
H5		2.538	-		
H6		2.207	muscle uptake		
H7		2.336	-		
H8		2.479	-		

**Figure A1:**
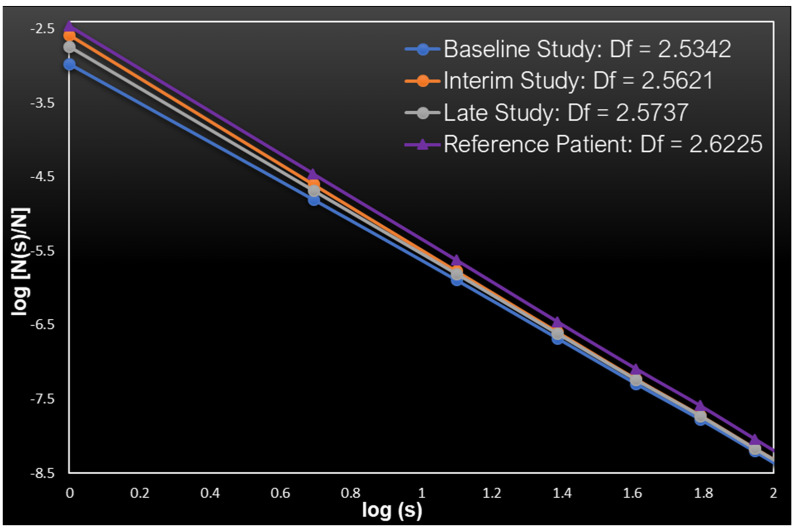
The normalized number of boxes **N(s)/N** which contain the tracer as a function of the box size **s** in a double logarithmic scale for patient P10, whose clinical diagnosis was PMR. The blue line corresponds to the baseline study of patient P10, the orange line to the interim study and the gray line to the final study of the same patient. For comparison, the purple line representing healthy control H3, is also depicted.

**Figure A2:**
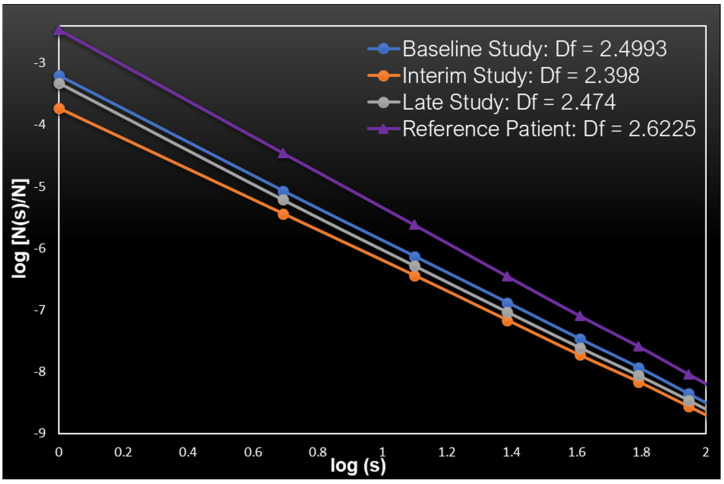
The normalized number of boxes **N(s)/N** which contain the tracer as a function of the box size **s** in a double logarithmic scale for patient P11, whose clinical diagnosis was PMD. The blue line corresponds to the baseline study of patient P11, the orange line to the interim study and the gray line to the final study of the same patient. For comparison, the purple line representing healthy control H3, is also depicted.

**Figure A3:**
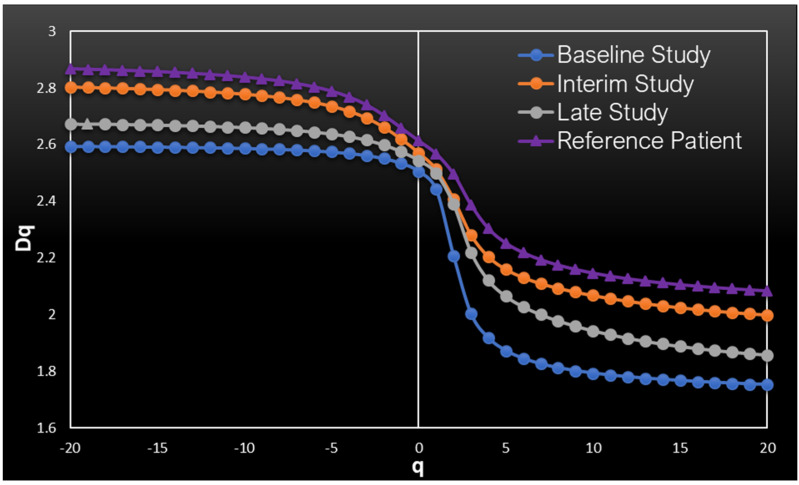
Typical MFS spectrum of patient with PMR. The blue line corresponds to the baseline study of patient P10, the orange line corresponds to the interim study and the gray line to the final study of the same patient. For comparison, the purple line representing healthy control H5 is also depicted.

**Figure A4:**
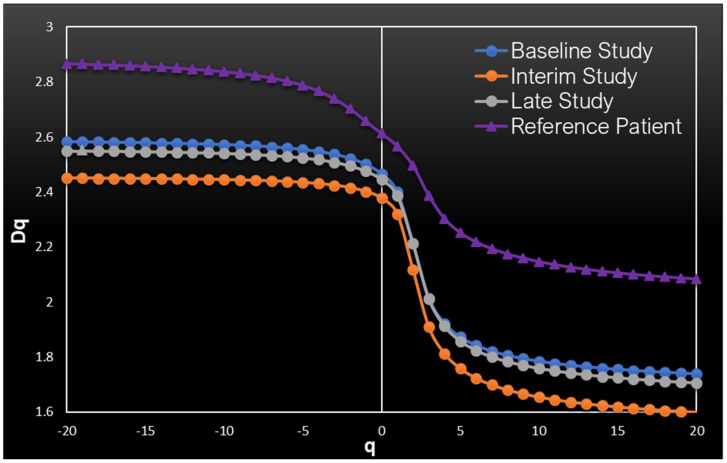
Typical MFS spectrum of patient with PMD. The blue line corresponds to the baseline study of patient P11, the orange line corresponds to the interim study and the gray line to the final study of the same patient. For comparison, the purple line representing healthy control H5 is also depicted.

## Data Availability

The authors confirm that the data supporting findings of this study are available within the article and upon request to the authors.

## Appendix A

Details on the calculations of the nonlinear measures FD, MFS and the cumulative multifractal measure are provided in the next three subsections.

### Appendix A1. Calculation of FD

After extracting the local ^18^F-FDG concentration ***C*(*i,j,k*)** for each patient at the three studies (Baseline, Interim, Final), we use the box-counting method as a measure of the FD on the C-matrices, which correspond to the PET-CTs images of the patients. Namely, we cover the image with 3D boxes of linear size **s** and calculate the number of boxes, **N(s)**, which contain any non-zero concentration of ^18^F-FDG. Note, that the actual amount of ^18^F-FDG is not important for the computation of FD and the calculation of the number of boxes **N(s)** is based on a binary (1 or 0) formula. Namely, a box of size **s** at position **(*i,j,k*)** is regarded as “containing ^18^F-FDG” in all cases where the concentration ***C*(*i,j,k*)** of ^18^F-FDG in this box takes any value greater than zero, ***C*(*i,j,k*)** **≥** **1**. Otherwise it is considered as “empty”, ***C*(*i,j,k*)** **= 0**. The tracer distribution in the patient body is governed by the following law [15,16]:

(A1) $$N\left(s\right)=Ms^{-D_{f}}\;$$

In Equation (A1), **M** is a constant related to the total concentration of the tracer in the body and **Df** is the value of the fractal dimension (FD). FD represents the dimensionality of the space covered by the tracer and if **Df** = 3, then the tracer is homogeneously distributed in the 3D space. If **Df** **<** 3, then the tracer covers a subspace with dimensionality smaller than the embedding dimensions. For the computation of **Df** the images (C-arrays) were divided in 3D boxes of linear sizes, in the range **s = 1 mm, 2 mm, 49 mm.** Larger boxes were not considered to avoid finite size effects (false effects or indications caused by limited statistics). When calculating the number of boxes with ***C*(*i,j,k*)** **≥** **1**, in some cases the body cannot be covered by an integral number of boxes. To take into account this mismatching, the number of boxes **N(s)** was normalized by the total number **N** of empty and non-empty boxes covering the structure. This normalization changes quantitatively the results, but does not cause any qualitative discrepancies.

To calculate the value of **Df** it suffices to plot **N(s)/N** versus **s** and to fit with a power law. The absolute value of the fitted exponent corresponds to **Df****.** Alternatively, one may plot **N(s)/N** versus **s** in a double logarithmic scale. In such a plot, the power law appears as a straight line whose tangent corresponds to **Df**.

### Appendix A2. Calculation of MFS

The MFS offers a more detailed description of the tracer dispersion because it takes into account not only the presence of the tracer in a binary form but also all the local concentrations as well as their moments. Call ***p*(*i,j,k*)** the normalized local concentration at position **(*i,j,k*)**, defined as:

(A2) $$p\left(i,j,k\right)=\frac{C\left(i,j,k\right)}{\sum_{i,j,k}C\left(i,j,k\right)}\;$$

In Equation (A2), ***p*(*i,j,k*)** can be regarded as the probability to find tracer at position **(*i,j,k*)**. In terms of probabilities, the generalized dimensions or moments, also known as MFS, are defined as follows:

(A3) $$\begin{array}{c} D_{q}=\frac{1}{q-1}\frac{ln\left[\sum_{i,j,k}p^{q}\left(i,j,k\right)\right]}{ln\left(s\right)}\;\mathrm{for}\;q\neq 1\\ D_{1}=\frac{\sum_{i,j,k}p\left(i,j,k\right)\;lnp\left(i,j,k\right)}{ln\left(s\right)}\;\mathrm{for}\;q=1 \end{array}\;$$

In Equations (A3), the index ***q*** takes all integer values **(–∞ ≤ *q* ≤ +∞)** and denotes the order of the generalized dimension, while the ensemble of all orders composes the MFS spectrum. While FD measures the presence of ^18^F-FDG locally on the images, without paying attention to the amount of ^18^F-FDG in each box, the MFS accounts exactly for all local concentrations of the tracer and thus gives more accurate account of the diagnosis and of the evolution of the disease.

For the calculation of MFS in Equation (A3), the limit of box sizes, **s** **→** **0,** is needed. For this reason, the smallest value, **s = 1 mm**, is used in the calculations.

### Appendix A3. Calculation of the Cumulative Measure *Δ*D(j)

It is worth noticing that the interpretation of the multifractal spectrum is more straight forward because the clinical picture (improvement or deterioration) of the patient between studies is mirrored on the relative position of the curves (and not on their slopes, as in the case of FD). To define a cumulative quantitative measure of clinical improvement or deterioration of the disease based on the MFS results, the average difference, **Δ*D*(*j*),** between ***D******_q_*** at the different stages ***j***, for **−10 ≤ *q* ≤ +10,** is used. This is defined as:

(A4)
$$\Delta D\left(j\right)=\frac{\sum_{q=-10}^{10}\left[D_{q}^{j}-D_{q}^{1}\right]}{21}\;\;$$

In Equation (A4) the index ***j*** takes the values ***j* = 2** or **3**. The quantity **Δ*D*(*j*)** defines the average difference of the twenty-one moments, **−10 ≤ *q* ≤ +10,** between the baseline study ***j* = 1** and the studies for ***j* = 2** or ***j* = 3**, after ICI treatment. If **Δ*D*(*j*) > 0** improvement of the patient outcome is recorded, while if **Δ*D*(*j*) < 0** deterioration is recorded. To allow for potential fluctuations around the ***D******q*** values, we use a threshold **u = 0.1** in this study, which defines the stable condition of the patient. Namely, if **|Δ*D*(*j*)| < u** the patient’s condition can be considered stable through treatments. Overall, the outcome for each patient after treatment, as presented in Section 3 and Table 3, is based on the above assumptions.

Equation (A4) can also be written as the difference between the mean spectra **<*MFS*>*****_j_***:

(A5)
$$\Delta D\left(j\right)=\frac{1}{21}\overset{10}{\underset{q=-10}{\sum }}D_{q}^{j}-\frac{1}{21}\overset{10}{\underset{q=-10}{\sum }}D_{q}^{1}=\langle MFS_{j}-MFS_{1}\rangle \;$$

As an additional approximation, in the present study the average ***MFS*** is taken over twenty-one moments, from **–10 ≤ *q* ≤ +10.**

## Appendix B

Typical calculations of FD for patients with PMR and PMD are presented below.

In Figure A1, it is noticeable that the slope (**Df**) of the baseline study is smaller than the one of the reference control. And, even after two (interim study) or four (final study) cycles of therapy the patient’s slopes remain below the reference’s slope (**Df** = 2.6225). Because there is an improvement of the interim and final study with regards to the baseline, this case is termed PMR.

In Figure A2, the slopes (**Df**) at the interim and final study stay smaller than the one of the first study, even after two or four cycles of therapy. Because there is no improvement and no stability of the patient’s condition, this case is termed PMD.

## Appendix C

Typical calculations of the MFS for patients with PMR and PMD are presented below. In the MFS it is easier to understand the evolution of the disease because the clinical improvement or deterioration of their disease is mirrored by the relative position of the curves (and not by their slopes, as in the case of FD).

In Figure A3, the MFS of patient P10 is presented at the three stages of PET-CT recordings. The MFS of the baseline study is considerably lower when compared to the reference control. After the first two cycles of medication the patients’ condition improves. This is mirrored in the MFS of the interim study (orange line) which levels closer to the reference. In the final study, after four cycles of medication, the patient’s condition slightly deteriorates but stays above the original baseline study. This patient is considered as PMR.

In Figure A4, we first note that the MFS spectrum of the patient P11 is considerably lower than the reference MFS, which is indicative of the diagnosed disease. After two cycles of treatment with monoclonal antibodies, at the interim study, the MFS (orange line) decreases, which points to clinical deterioration. After four cycles of treatment, at the 3rd study, the MFS improves slightly, but stays lower than the baseline study. This condition is consistent with PMD, because the patient’s condition does not improve with respect to the initial baseline diagnosis, neither in the interim study nor at the final one.
